# Resuscitation After Hemorrhagic Shock in the Microcirculation: Targeting Optimal Oxygen Delivery in the Design of Artificial Blood Substitutes

**DOI:** 10.3389/fmed.2020.585638

**Published:** 2020-10-27

**Authors:** Carlos Munoz, Federico Aletti, Krianthan Govender, Pedro Cabrales, Erik B. Kistler

**Affiliations:** ^1^Department of Bioengineering, University of California, San Diego, La Jolla, CA, United States; ^2^Department of Anesthesiology and Critical Care, University of California, San Diego, La Jolla, CA, United States; ^3^Department of Anesthesiology and Critical Care, Veterans Affairs San Diego Healthcare System, San Diego, CA, United States

**Keywords:** hemorrhagic shock, resuscitation, microcirculation, blood substitutes, viscosity, oxygen delivery, shear stress

## Abstract

Microcirculatory preservation is essential for patient recovery from hemorrhagic shock. In hemorrhagic shock, microcirculatory flow and pressure are greatly reduced, creating an oxygen debt that may eventually become irreversible. During shock, tissues become hypoxic, cellular respiration turns to anaerobic metabolism, and the microcirculation rapidly begins to fail. This condition requires immediate fluid resuscitation to promote tissue reperfusion. The choice of fluid for resuscitation is whole blood; however, this may not be readily available and, on a larger scale, may be globally insufficient. Thus, extensive research on viable alternatives to blood has been undertaken in an effort to develop a clinically deployable blood substitute. This has not, as of yet, achieved fruition, in part due to an incomplete understanding of the complexities of the function of blood in the microcirculation. Hemodynamic resuscitation is acknowledged to be contingent on a number of factors other than volume expansion. The circulation of whole blood is carefully regulated to optimize oxygen delivery to the tissues *via* shear stress modulation through blood viscosity, inherent oxygen-carrying capacity, cell-free layer variation, and myogenic response, among other variables. Although plasma expanders can address a number of these issues, hemoglobin-based oxygen carriers (HBOCs) introduce a method of replenishing the intrinsic oxygen-carrying capacity of blood. There continue to be a number of issues related to HBOCs, but recent advances in the next-generation HBOCs show promise in the preservation of microcirculatory function and limiting toxicities. The development of HBOCs is now focused on viscosity and the degree of microvascular shear stress achieved in order to optimize vasoactive and oxygen delivery responses by leveraging the restoration and maintenance of physiological responses to blood flow in the microcirculation. Blood substitutes with higher viscous properties tend to improve oxygen delivery compared to those with lower viscosities. This review details current concepts in blood substitutes, particularly as they relate to trauma/hemorrhagic shock, with a specific focus on their complex interactions in the microcirculation.

## Introduction

Strategies for reperfusion after hemorrhage are typically aimed at the restoration of systemic hemodynamic indices such as blood pressure, heart rate, and venous return. Commensurate improvements in organ function such as mental status and urine output serve as crude confirmation that end-organ perfusion is preserved. However, the mechanisms that determine survival and recovery after hemorrhagic shock ultimately occur in the microcirculation, a milieu of which there is little understanding and no direct ability to control or modulate. There exists, therefore, a disconnect between the clinical interventions that are applied to treat hemorrhagic shock and the downstream consequences of these interventions in the microcirculation (1).

Microvascular dysfunction associated with hemorheological alterations results in oxidative stress, release of proinflammatory cytokines (2), and disruption of endothelial integrity (specifically the endothelial glycocalyx). Given the importance and delicate nature of the endothelium integrity and its physiological function in the microcirculation, its protection may prove a promising target in critical care settings. Previous *in vitro* studies showed that shear significantly increased glycosaminoglycan synthesis in endothelial cells (3). Fluid shear stress on the endothelium modifies the organization of inter-endothelial junctions (4) and can alter the barrier properties of the endothelium by activating intracellular signaling events (3–5). The glycocalyx represents a major barrier against the extravasation of fluids and colloids; sieving and binding of intravascular colloids from the plasma restrict transport of other plasma molecules (6). Thus, the endothelial glycocalyx is better preserved when the appropriate fluid is used during resuscitation from hemorrhagic shock (HS).

Fluid resuscitation is used to restore/preserve blood volume. Colloidal resuscitation fluids stabilize hydrostatic capillary pressure, which is required to preserve functional capillary density (FCD) (7). FCD determines the serum lactate levels and acid–base balance and is critical for tissue survival after HS (8). We demonstrated that the maintenance of adequate FCD is directly related to capillary pressure, which can be preserved by increasing plasma viscosity using high-viscosity resuscitation fluids (9). Hemodilution with high-viscosity resuscitation fluids (with viscosities of 4 cP) does not increase diluted blood viscosity above that of undiluted whole blood, but it increases plasma viscosity and endothelial wall shear stress, thus promoting the production of shear stress-mediated vascular autocoids [e.g., nitric oxide (NO) and prostaglandins] (10). These autocoids cause vasodilation and increase microvascular blood flow, which increases oxygen (O_2_) delivery without increasing the O_2_-carrying capacity (11).

The cornerstone for resuscitation after hemorrhage is blood, either whole blood as is now increasingly being practiced in both military and civilian trauma settings (low-titer type O-positive whole blood) or, more commonly, blood component therapy in the form of packed red blood cells (PRBCs), fresh frozen plasma (FFP), and platelets. The critical issue with the use of blood as the first line of treatment in trauma and hemorrhagic shock (T/HS) is its availability from donors. In an effort to develop usable alternatives, artificial blood substitutes—tacitly defined as alternatives to red blood cells (RBCs), not plasma or platelets—have been studied for many years. A major barrier to progress in this area has been the lack of a mechanistic understanding of the processes associated with oxygen transport by blood and the physiological interaction of blood and the tissue as a system in the microcirculation.

Blood substitutes have the potential to be engineered in such a way as to replicate or even optimize the critical biophysical parameters of circulating blood, enabling the production of life-sustaining product for critical patient needs. In order to develop a successful blood substitute for T/HS, however, an improved understanding of the role of blood is necessary.

The most important biophysical parameter of the interaction between blood and tissues at the microvascular level is blood viscosity, which is significantly altered in hemorrhagic shock because of hemodilution and reduced hematocrit, with a direct impact on microvascular function.

In order to design efficient blood substitutes able to fulfill the metabolic demands of tissues during resuscitation, key alterations induced in the microcirculation by T/HS should be analyzed from the point of view of their impact on viscosity. Therefore, optimizing viscosity should become the new paradigm in the design of next-generation blood substitutes.

## Transfusion Medicine: Mimicking The Blood to Preserve Oxygen Delivery to The Tissues

Demand for whole blood and components is presently satisfied by donor blood, a source that is projected to become increasingly scarce and ultimately insufficient for healthcare needs as the proportion of older individuals increases in the United States and worldwide (12). Advances in recombinant technology and stem cell manipulation may be able to correct some of the projected deficits in blood product inventories; however, the technology required to produce a useable product in the quantities needed is not presently available at costs affordable by the health care system (13).

Although component therapy is much more versatile than whole blood resuscitation, which is hampered by shorter storage times (21–35 days depending on anticoagulant, e.g., citrate phosphate dextrose vs. citrate phosphate dextrose adenine) because of the necessary dilution of blood factors for storage, resuscitation at the recommended 1:1:1 ratio (PRBCs/FFP/platelets) results in a resuscitation fluid diluted to a hematocrit (Hct) of ~29%, platelet concentration of 88,000/mcl, and 65% of the normal coagulation factor activity, all substantially inferior to component concentrations in whole blood. Of potential importance is that the viscosity, as well as the oxygen-carrying capacity of the 1:1:1 ratio, is markedly reduced compared to that of whole blood.

Blood substitutes are engineered as resuscitation fluids which ideally mimic the main functions of biological blood to target hypoxic tissue caused by shock. Because oxygen delivery to tissues has historically been considered to be the goal of blood substitutes, most research done on blood alternatives has been hemoglobin (Hb) based. Blood substitutes that leverage Hb as the method to sustain the oxygen-carrying capacity (O_2_CC) are known as hemoglobin-based oxygen carriers (HBOCs). However, circulating cell-free Hb has been associated with a number of adverse outcomes, including inflammation, thrombosis, oxygen free radical generation via its heme moiety, kidney failure through glomerular compromise, and hypertension and vessel constriction NO scavenging (14). In fact, the U.S. Food and Drug Administration (FDA) stipulates that any proposed blood substitutes specifically address the issue of NO scavenging as part of their approval process.

To circumvent limitations inherent using free Hb as a blood substitute, several different variations of HBOCs have been designed, including alpha-alpha cross-linked Hb (ααHb) (15), polyethylene glycol-bound Hb (PEG-Hb) (16), and polymerized hemoglobin (polyHb) (17). However, none of these has been proven effective as a replacement for blood or blood products. Non-Hb-containing modalities have been studied as well, such as perfluorocarbons (PFCs), a class of compounds that readily bind both oxygen and carbon dioxide. The discovery that PFCs could dissolve a comparatively large amount of oxygen, albeit at high oxygen partial pressures, suggested using this vehicle as blood substitute. PFCs have a number of potential advantages over HBOCs, most notably their lack of potential infectivity and their ability to be mass produced as synthetic compounds (18). However, the use of PFC-based blood replacement fluids has not materialized, in part because of the lack of definitive experimental studies on the physiology related to altered blood physical properties and changes in the distribution of oxygen partial pressures in the circulation.

To date, there are virtually no viable alternatives to hemoglobin for oxygen transport from the lungs to the tissues because of the ability of hemoglobin to bind a large amount of oxygen *via* a chemical reaction. Thus, hemoglobin is still the key constituent of presently designed oxygen-carrying blood substitutes. Various modifications and formulations continue to optimize its performance and have largely eliminated inherent toxicities, but continued development of HBOCs requires addressing the underlying issues of microcirculatory dysfunction, especially during T/HS.

## Microvascular Pathophysiology In Hemorrhagic Shock

The blood that circulates through the body conducts a number of functions that are essential for survival, such as perfusing tissues with oxygen, collecting waste products of metabolism, and maintaining an acceptable pH via CO_2_ offloading. In the event of severe blood loss, the intrinsic O_2_CC of the microcirculation begins to falter as the concentration of RBCs declines. The drop in blood pressure caused by hemorrhage results in a fall of the hydrostatic pressure at the arteriolar end of the capillaries. This reduced pressure head affects fluid movement across the capillary bed, promoting reabsorption. With the pooling of fluid in the vasculature to maintain fluid flow, the concentration of RBCs is further diluted. A myogenic response takes place, causing an inward remodeling of the vessels and vasoconstriction (9). There are many local regulatory mechanisms that respond during hypovolemic shock in a tissue-dependent manner. For example, adenosine and prostanoids together are responsible for most of the dilation in femoral resistance vessels (19), while the myogenic response is dominant in other tissues like the kidney (20). While local flow regulatory mechanisms are important determinants of blood flow to individual organs, the central nervous system also exerts an important influence through sympathetic nerves in most organs (21). Some of the most striking changes seen at the tissue level due to severe shock are a reduction in O_2_CC, a decrease in blood viscosity, a decrease in vessel wall shear stress (WSS), shedding of the protective glycocalyx barrier (22), and pathologic hyperfibrinolysis and diffuse coagulopathy (23–25).

T/HS also takes a toll at the cellular level. With the decrease in tissue perfusion, anaerobic metabolism becomes the key source of energy production. Diminished O_2_ delivery to cells results in the shunting of pyruvate to increase lactate production at the expense of oxidative phosphorylation. Since cellular respiration is the key metabolic process that produces ATP to fuel tissues and a lack of oxygen shunts energy production anaerobically, a primary metabolite is lactate, whose plasma levels rise in consequence of this metabolic alteration. Inadequate aerobic cellular respiration ultimately leads to mitochondrial dysfunction (26). As ATP supplies dwindle, cellular homeostasis ultimately fails, and cell death ensues through necrosis from membrane rupture, apoptosis, or necroptosis.

### Oxygen Debt

The decreased rate of O_2_ delivery results in tissue “oxygen debt” (24). If this debt is not “paid,” the tissue will not survive. Therefore, at the microvascular and cell level, three principles must be considered to devise an effective microcirculation-targeted resuscitation strategy: (1) prevention of “oxygen debt” accumulation, (2) repayment of “oxygen debt,” and (3) minimization of the time to “oxygen debt” resolution (27).

A major challenge of resuscitation is determining oxygen delivery to the tissues (DO_2_) and oxygen consumption by the tissues (VO_2_). Serial lactate measurements coupled with central venous oxygen saturation (SVO_2_) can aid in this determination on the macro level. However, these parameters do not distinguish between microcirculatory arterial–venous shunting and true increases in perfusion or between lactate generation vs. uptake. At present, there is no reliable method to clinically measure regional tissue oxygenation, particularly in organs that are not amenable to direct examination (28–30).

### Microcirculation Analysis as a Tool to Design Novel Resuscitation Strategies

Systemic parameters such as heart rate and blood pressure have historically been used to monitor recovery from shock. However, if we consider the idea of “oxygen debt” and the use of DO_2_/VO_2_ to aid in our understanding of the reoxygenation of previously ischemic tissue, it becomes apparent that there must be a focus on the microcirculation in addition to the restoration of systemic indices. Microcirculatory integrity is the principal determinant for tissue oxygenation, nutrient supply, organ function at the tissue level, and adequate immunological function (31).

### Hamster Models for the Analysis of Microcirculation in Hemorrhagic Shock

There are a variety of animal models used to understand the microcirculation in shock; one of the most common is the hamster model (32). Hamsters have a low central partial pressure of oxygen (PaO_2_) of ~57 mmHg, corresponding to a Hb O_2_ saturation of 84% (32). Because the baseline arteriolar partial pressure of oxygen (pO_2_) is so low, calculation of the pre-microvascular oxygen consumption shows that this species is very efficient at delivering oxygen to the tissues, and very little oxygen leaves the circulation before delivery to the microcirculation, with the change in blood saturation of only about 3% (33).

While in clinical practice the sublingual area has been typically used to measure microvascular performance using dark-field microscopy, observation of the microcirculation in hamsters is commonly achieved via intravital microscopy of the dorsal skin flap: the skin is lifted, creating a skin fold, which is supported by two titanium frames with 12-mm circular openings. One frame is sutured on one side of the skin fold. The opposite skin layer is removed following the outline of the window, leaving only a thin layer of retractor muscle, connective tissue, and intact skin. The exposed tissue is then sealed with a glass cover held by the other frame, creating an environment to make optical measurements of the microcirculation *in vivo* (33–35). Two vessels are generally cannulated, the carotid artery for monitoring blood pressure and the femoral or jugular vein for the infusion of fluids and dyes. Intravital microscopy enables better understanding of the inputs that determine the cellular and subcellular processes within multiple organs. While this type of microscopy can only be implemented in hamsters and other rodents, and does not directly translate to humans, the observed intrinsic regulatory processes at the tissue level do not differ between humans and rodents. This experimental setup allows for microhemodynamic measurements of blood velocity using the photodiode cross-correlation technique and vessel diameters *via* video image shearing (36, 37). The study of flow in the microcirculation enables an improved understanding of the mechanisms underlying oxygen delivery (38). Microvascular pressure and the perfusion of blood in the tissues can be quantified by FCD or the number of perfused capillaries per unit area (9). Capillaries are considered functional if RBCs transit though the capillary segments during a prescribed unit of time. Capillary perfusion is necessary for the oxygenation of the tissues and, perhaps as importantly, the removal of metabolic waste. High-resolution microvascular pO_2_ measurements can be made using phosphorescence quenching microscopy (PQM) or hyperspectral imaging. PQM is a technique based on oxygen-dependent quenching of phosphorescence emitted by an albumin-bound metalloporphyrin complex after light excitation (39). Hyperspectral imaging is a technique that utilizes the spectra differences in oxyhemoglobin and deoxyhemoglobin to determine oxygen saturation in the microcirculation (40).

**Figure 1:**
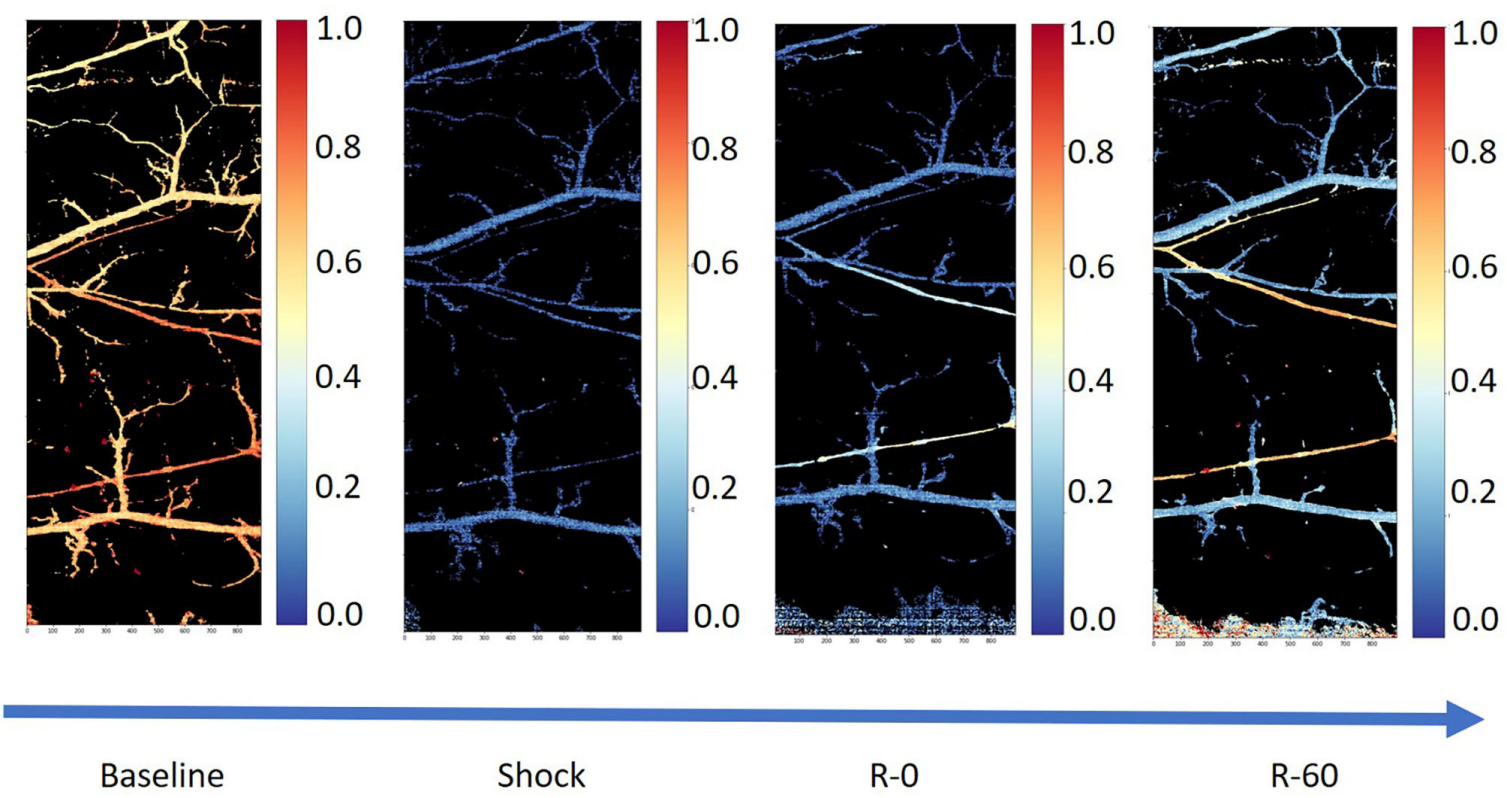
Tissue perfusion in the microcirculation using hyperspectral images from the skin pouch of a Golden Syrian hamster following hemorrhage (50% total blood volume) and a resuscitation using whole blood (25% total blood volume in 5% albumin; Sigma-Aldrich, Saint Louis, MO). The first image (from left to right) is the microcirculation at baseline, the second one is the microcirculation in shock, the third one is the microcirculation immediately after the completion of resuscitation, and the fourth and final image is the microcirculation 60 min post-resuscitation.

Figure 1 shows a typical example of tissue perfusion in the skin pouch of a Golden Syrian hamster during experimental hemorrhage obtained using hyperspectral imaging. The experiments were approved by the Institutional Animal Care and Use Committee of the University of California, San Diego, and performed following the NIH Guide for the Care and Use of Laboratory Animals, 8th edition (2011). The images were acquired using a Pika-L hyperspectral imaging system with a linear translation stage (Resonon, Bozeman, MT) with a spectral range of 390–1,020 nm, a spectral resolution of 2.1 nm, and a spatial resolution of 900 pixels per line. Each image consisted of a 2,900 × 900 × 300 hypercube. After the acquisition, the images were truncated and resampled in the spectral domain between 500 and 590 nm. Calculation of the relative deoxygenated and oxygenated hemoglobin abundances were completed based on a calibration standard for hemoglobin using Beer's law.

When quantifying microcirculatory dysfunction resulting from hemorrhagic shock, there are several variables that must be considered: the dynamic changes in flow, vasoactive responses, the tissue pO_2_ in the microcirculation, lactate concentration, etc. Regardless of the hemorrhagic shock model (fixed pressure vs. fixed volume), microcirculatory parameters are used as barometers of successful resuscitation interventions (41–43). However, there is no consensus on a quantitative definition of “recovery.” The literature commonly refers to three schools of thought in defining microvascular recovery after hemorrhagic shock: (1) increased microvascular viscosity (33–35), (2) increased microvascular pressure (44, 45), and (3) improved hemodynamics in the microvasculature (46). Arguments have been made for each parameter individually, but they all hinge on what is defined as “recovery,” which should be taken into consideration for the optimization of blood substitutes design.

## Local Regulation of Blood Flow and Oxygen Delivery

Engineering any type of blood substitute requires an understanding of the complex interactions between oxygen consumption, changes in the composition of blood and its viscosity, and the oxygen dissociation curve for hemoglobin. A decrease in O_2_CC due to hemodilution induces vasodilation, and the restoration of the lost RBCs with an oxygen carrier can produce additional signals depending on whether the carrier has a high or low affinity for oxygen. Facilitated release of oxygen from blood is a property defined by the value of pO_2_ at which blood is 50% saturated (P_50_). A high oxygen affinity (low P_50_) results in oxygen being preferentially unloaded in the capillaries, and vice versa (32).

An important consideration when designing blood substitutes that will necessarily unload oxygen in the microcirculation is oxygen consumption by the vessel wall itself. Several studies have concluded that oxygen consumption may be extraordinarily high at the vessel wall and increases during vasoconstriction (47, 48). The marked uptake of oxygen at the vessel wall by vascular smooth muscle is somewhat analogous to the significant increases in oxygen use by the skeletal muscle during exercise; vasoconstriction of vessel beds necessarily requires energy and, thus, increased oxygen consumption.

In a reciprocal fashion, increases in circulating pO_2_ result in increased vessel vasoconstriction. The reasons for this are unclear, but changes in vessel wall oxygen uptake (and thus vascular tone) in response to circulating pO_2_ suggest that the purpose of vasoconstriction in the presence of elevated circulating pO_2_ is to limit tissue pO_2_ to within a fairly narrow range. This phenomenon further complicates the engineering of effective HBOCs for treatment during T/HS as shock induces vasoconstriction, indicating a potential need to satisfy the vessel wall's oxygen debt before oxygen can enter the tissue beds.

Blood substitute design is often tested using extreme hemodilution, a condition in which systemic Hct is reduced to the point where oxygen delivery becomes dependent on the intrinsic oxygen-carrying capacity of the circulation. This approach allows evaluating blood substitutes characterized by high-affinity hemoglobin products such as 4% Mal–PEG–hemoglobin (MP4) (Hemospan, Sangart Inc., San Diego, CA) or low-affinity bovine-derived PolyBvHb (PBH) (Biopure Inc., Boston, MA) (Table 1).

**Table 1:** Typical properties of hemoglobin (Hb)-based oxygen carriers vs. blood/plasma.

	**Concentration (g/dl)**	**Viscosity (cp)**	**COP (mmHg)**	***P*_**50**_ (mmHg)**
Hemospan^a^ (MP4)	4.2	2.5	55	6
Oxyglobin^b^ (PBH)	13.2	1.8	40	54
Blood		4.2	20	32
Plasma		1.2	20	

*COP, colloid osmotic pressure; P_50_, pO_2_ at which blood is 50% saturated*.

a *Polyethylene glycol-conjugated Hb, Sangart Inc., San Diego, CA*.

b *Polymerized bovine hemoglobin, Biopure Corp., Boston, MA*.

The goal of high-affinity Hb-based blood substitutes is to create a reservoir of oxygen that only unloads oxygen when the circulating blood arrives at tissue regions where the pO_2_ is very low, promoting oxygen delivery through diffusion. However, if the affinity for oxygen is too high, the inability to release oxygen may become problematic, leaving the tissue hypoxic. On the other hand, in low-affinity Hb-based blood substitutes, a rightward shift in the O_2_ dissociation curve should theoretically increase O_2_ extraction and improve maximal O_2_ uptake. This manipulation of the P_50_ offers the opportunity to vary O_2_ delivery to the tissues without altering blood flow or arterial O_2_ content, promoting oxygen delivery *via* convection rather than diffusion.

Blood viscosity is a critical determinant of tissue perfusion because of its direct influence on vascular resistance. Blood viscosity depends primarily on RBC concentration (hematocrit) and secondarily on the viscosity of plasma. Restoration of viscosity in the initial resuscitation period is a common method for assessing recovery in the microcirculation after hypovolemic shock (33, 42, 43); it is not, however, widely studied clinically. As an alternative to oxygen-carrying modalities, restitution of blood loss with non-oxygen-carrying plasma expanders (PEs) can be effectively and safely accomplished after up to a 50% loss of the RBC mass using fluids with plasma-like viscosity, which induce a compensatory increase in cardiac output aimed at maintaining oxygen delivery (49). Additionally, interventions that increase the viscosity of circulating blood after shock can mitigate the effects that reduced viscosity have on mechanical transduction of the endothelium, potentially preserving the native myogenic responses that normally regulate microvascular flow and pressure (50). Large-molecular-diameter HBOCs play a similar role to PEs, increasing blood and plasma viscosity and preserving microvascular perfusion (51).

**Figure 2:**
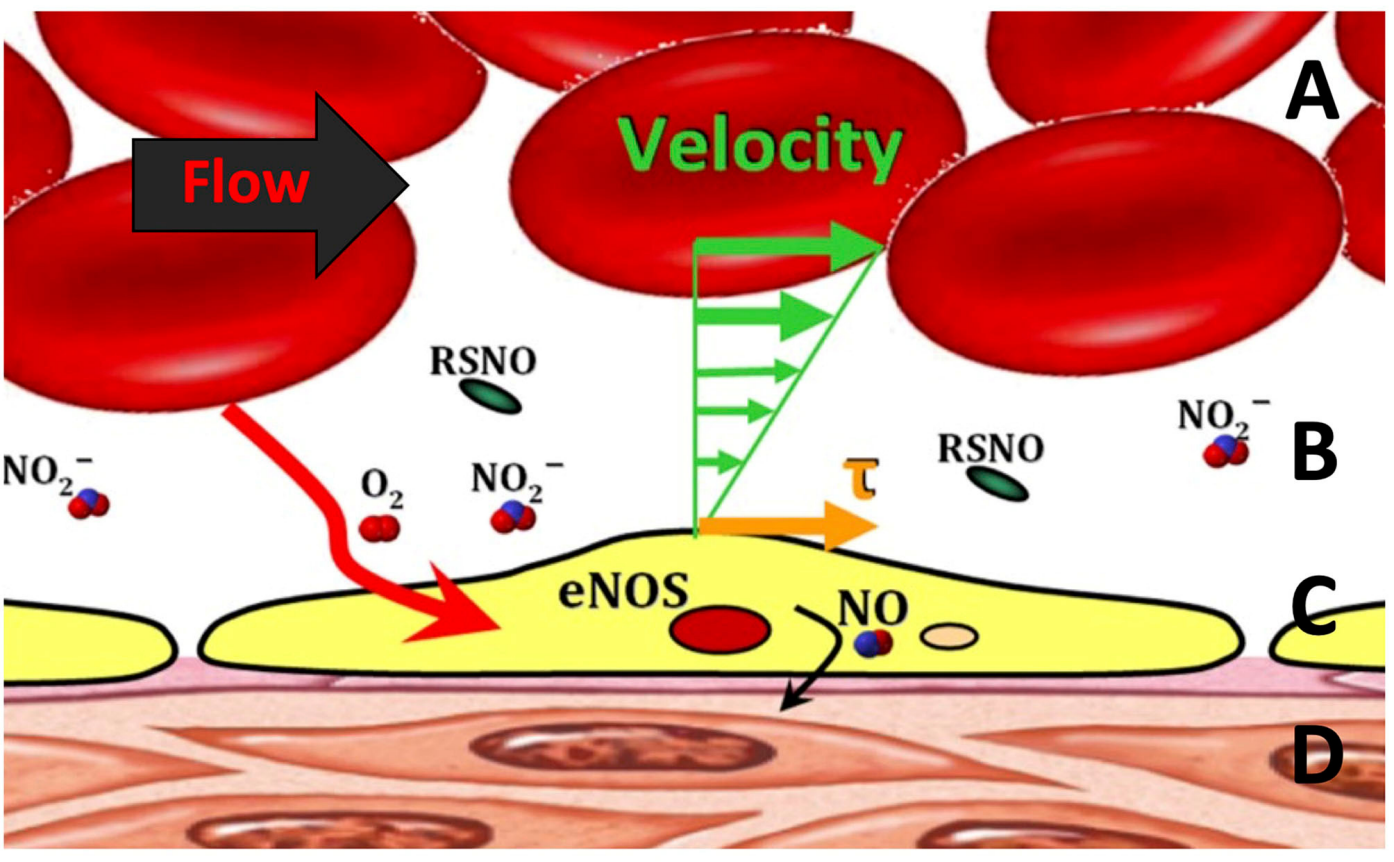
Blood flow through the microcirculation. **(A)** The bulk of red blood cell (RBC) flow, which is related to the shear stress (τ). **(B)** Cell-free layer in the microcirculation. **(C)** Endothelial cell lining generating endothelial nitric oxide synthase (eNOS), *S*-nitrosothiols (RSNO), and nitric oxide (NO) *via* mechanotransduction. **(D)** Smooth muscle layer encapsulating the entire vessel.

Microvascular perfusion and FCD are controlled in part through shear-mediated factors. For example, shear stress exerted on the blood moving near the endothelial surface releases dilatory autocoids (7, 52, 53). Increasing the shear stress on the endothelial surface promotes the expression of anti-inflammatory, antiproliferative, anti-apoptotic, and antioxidative genes, all of which reduce the effects of systemic inflammation associated with hemorrhage shock (54). Recent studies have demonstrated a new, microscopic area of the cell-free layer (CFL) which appears to be the critical focal point of the interaction between blood and microcirculation (Figure 2) (55). The CFL regulates oxygen transport and capillary perfusion as it determines the distance between the circulating oxygen supply and the tissue as well as the degree of shear stress on the endothelial wall caused by the passing blood.

Formation of a wider cell-free layer reduces effective blood viscosity near the vessel wall and, thus, the amount of shear experienced by the endothelium, a principal stimulus for release of the potent vasodilator NO (56, 57). However, the CFL has been shown to act as a barrier to NO scavenging, potentially mitigating the effects of reduced shear (58). The CFL is also an important determinant of the rate of oxygen diffusion from the red cells to the tissues. The radial diffusion gradient of oxygen from the hemoglobin carried by RBCs transits from blood to the parenchymal cells through several barriers including plasma, the endothelial layer, and interstitial fluid. Due to the low solubility of oxygen in plasma, the width of the cell-free plasma layer barrier significantly limits oxygen delivery (59, 60). Thus, the interplay between the CFL and the microcirculation serves to regulate vascular perfusion via changes in NO scavenging and oxygen delivery to the tissues. The initial response of the vasculature during T/HS is to vasoconstrict, thus reducing the CFL and thereby potentially increasing NO bioavailability and oxygen delivery to the tissue.

An improved understanding of the fundamental role of the CFL in the microcirculation has contributed to assessing how the circulation responds to small (±10%) changes in Hct and blood viscosity (61). A novel methodology to measure the CFL width (62–64) has been recently developed. This technique is based on high-speed video recording (up to 30,000 frames/second) and a thresholding algorithm that converts the interface between blood and plasma into a black and white image, defining the stochastic surface of the RBC column and identifying the location of the plasma/vessel wall interface. Studies of the trajectories of RBCs at the blood column/plasma interface have identified several features of relevance, particularly the RBC exclusion zone which outlines the glycocalyx. Studies using this technique have led to conclude that CFL width is a function of Hct (65). CFL thickness is an important feature of blood flow in the microcirculation and is proportional to the shear stress and the thickness of the endothelial glycocalyx. Hemorrhage alters glycocalyx structure and function, and changes in the CFL thickness can reflect glycocalyx shedding (64).

**Figure 3:**
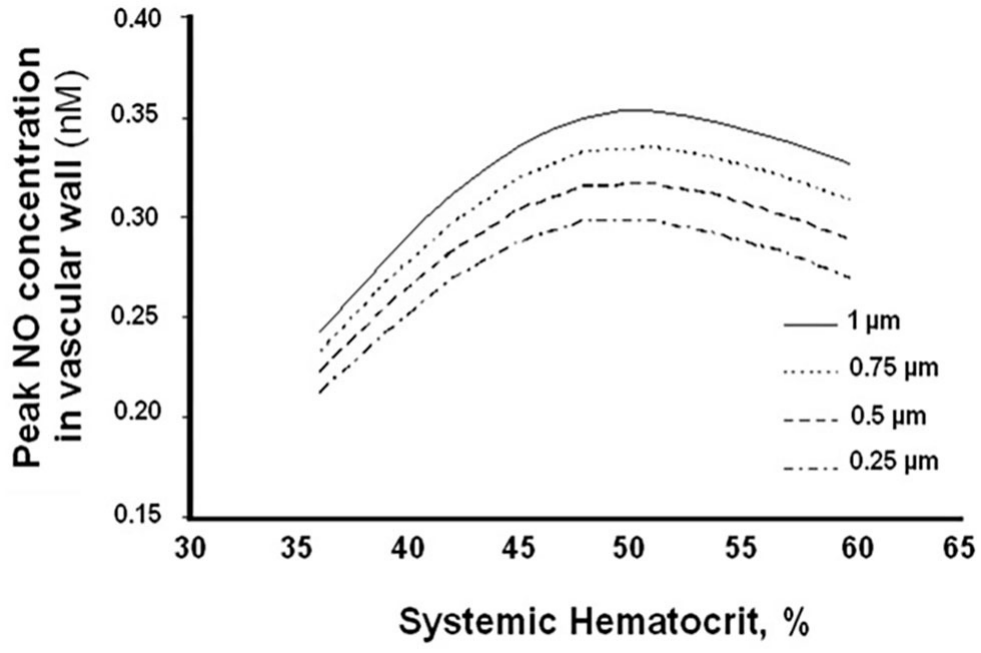
Arteriolar wall nitric oxide bioavailability as a function of systemic hematocrit percentage for different cell-free layer (CFL) widths.

Further, small changes in Hct result in large changes in the CFL, significantly affecting arteriolar wall NO bioavailability and wall shear stress (WSS) (Figure 3) (61). This relationship between Hct and CFL, described by mathematical modeling, confirms previous assumptions that (1) NO bioavailability in hemodilution increases by decreasing CFL width; (2) hemodilution decreases Hct and plasma viscosity, lowering WSS and increasing CFL width; and (3) FCD is directly proportional to WSS in hemodilution.

Previous studies measuring CFL width changes after 40% hemodilution using low-viscosity PEs (e.g., albumin solutions) demonstrate small but measurable decreases in CFL width. It is hypothesized that this effect is significantly reduced with high-viscosity PEs because of their comparatively large hydrodynamic radius compared to albumin. Furthermore, mathematical modeling of the CFL width effects suggests the hypothesis that the width is in part determined by the type of colloid used (61). One of the relevant processes may be shear-dependent dilation, which provides a dispersive lateral force to the direction of flow. The assumption is that this force acts on the flowing RBCs, thus confirming the importance of viscosity in microcirculation hemodynamics and function.

## Microcirculation Function and Blood Substitute Viscosity

Survival in hemorrhage after ensuing extreme hemodilution is primarily dependent on the maintenance of FCD and secondarily on tissue O_2_. In the previous section, CFL width was introduced as the key parameter of wall shear stress modulation in the microcirculation. As the bioavailability of NO is inversely proportional to CFL width, NO-induced vasodilation is the main determinant of FCD and microvascular perfusion. Based on these observations, increasing wall shear stress in the CFL after T/HS should improve perfusion by the combination of three effects: (1) decreasing plasma CFL width; (2) increasing plasma viscosity; and (3) increasing blood vessel flow velocity. Solely increasing plasma viscosity has potential limitations because, in conditions of extreme hemodilution after hemorrhage, resuscitation with a high-viscosity plasma expander increases the workload of the heart while a simultaneously lower Hct limits O_2_ delivery. It is assumed, however, that this intervention can increase the width of the blood column and (moderately) the resistance to plasma fluid flow in the glycocalyx, which would decrease effective CFL width. Current understanding of this complex dynamic suggests that developed blood substitutes should thus be of high viscosity (51).

### Microvascular Pressure

Modulating microvascular pressure following hemorrhagic shock directly relates to FCD, a key determinant of survival from T/HS. During shock, the microvascular pressure plummets, resulting in a sharp decrease in the number of perfused capillaries with implications for the surrounding tissue. Adequate perfusion of the capillary bed is thus vital as it is associated with oxygen delivery (diffusion) to the tissue from the systemic circulation as well as the continuous washout of metabolites (31). As tissues become hypoxic, they also become acidotic and lactate is accumulated. This, in turn, negatively affects systemic pH and base excess. The severity of the changes seen in arterial blood gas measurements (including pH, PaO_2_, PaCO_2_, base excess/deficit, and lactate) can be used to assess the extent of shock (34, 66). Increasing FCD restores flow to the tissues, alleviating local as well as systemic acidosis as oxygen diffusion into the tissue increases, shifting metabolic processes once again toward aerobic metabolism. Increasing the microvascular pressure may be accomplished systemically by the use of resuscitation fluids that increase plasma viscosity or have high colloid osmotic pressure (COP) and are therefore able to draw fluid into the microvasculature.

### Microvascular Flow

Peripheral hemodynamics are a typical measure of recovery in experimental T/HS (31). In the quiescent state, microcirculatory hemodynamics are equilibrated to the point where RBC velocity ensures the optimal diffusion of oxygen to the tissues through convective processes. In shock, flow can be significantly impaired and reduced dramatically, resulting in inadequate tissue oxygenation. Thus, increasing the intravascular microcirculatory volume *via* resuscitation fluid will enhance flow and tissue (re)oxygenation (67). Regardless of the mechanism for reperfusion, microvascular recovery following shock is contingent on how well oxygen delivery to previously hypoxic tissues can be achieved.

## Conclusions

Plasma expansion and blood transfusion are major medical interventions in T/HS, and even minor improvements in their efficacy will have significant healthcare repercussions. Advancements from current research will help to guide the development of fluids that are safe and effective blood substitutes. These may then lead to the development of transfusion protocols that include O_2_ transport indices and blood viscosity as targets of an effective microcirculation-targeted resuscitation.

Experimental results from microcirculation studies modeling blood loss and blood transfusion suggest that the present focus on restoring blood volume and O_2_-carrying capacity should be redirected toward using fluids that trade the requirement for O_2_-carrying capacity with the enhancement of microcirculatory O_2_ delivery. It then follows from this hypothesis that the development of artificial blood substitutes, which are presently aimed at restoring the blood O_2_-carrying capacity, should rather be designed to restore blood viscosity. This corresponds to a fundamental change in the perception of what blood has to accomplish in the circulation, which includes the maintenance of a shear stress environment conducive to adequate bioavailability of NO as a function of Hct with a proportional relationship to the CFL. In this new view, artificial blood substitutes for resuscitation after hemorrhagic shock should be optimally designed with the following priorities in mind: restoration of circulating (1) volume, (2) viscosity, and, lastly, (3) O_2_-carrying capacity.

A potential clinical implication might revolve around the question “What is the patient's blood viscosity?” in the assessment of a hemorrhaging victim, with the answer to this question used as a guide to the fluid treatment strategy. Theoretically, demand for rapid and reliable blood viscometry would be expanded, and the use of blood transfusions substantially diminished, to eventually be relegated to extreme conditions. Determining the blood viscosity that optimizes systemic and microvascular function when Hb is reduced beyond the transfusion trigger is a challenge. We have extensive experience in the analysis of shock resuscitation using a variety of blood substitutes, besides blood (32, 33, 41, 42, 65, 68–81). However, the definitive test of the viscogenic hypothesis requires its verification in T/HS, one of its most likely areas of application.

Hemorrhagic shock entails a complex series of cascading events that have synergistic pathophysiologic effects, from the cellular level up to the entire cardiovascular circuit as a whole. Theories that revolve around rheology, functional physiology, and hemodynamics have been proposed regarding the optimal way to successfully treat a patient in shock. Vasopressor and inotrope therapies have been explored as part of the resuscitation strategy from hemorrhagic shock, either as main treatments or simultaneously in combination with fluid support, with mixed success. While vasopressors and inotropes could improve systemic hemodynamic metrics representative of the effectiveness of resuscitation (for instance mean arterial pressure), their benefit to the blood flow in the microcirculation is questionable. Indeed, they could have a limited beneficial effect or even contribute to jeopardize further the long-term recovery of the microcirculation from hemorrhagic shock, especially during severe hypovolemia, when venous return is insufficient to support systemic O_2_ requirements. All of these hypotheses relate back to replenishing the pool of oxygen that was depleted in the tissues. Arguments can be made for each of these hypotheses. However, artificial blood substitutes must satisfy all three hypotheses to be a viable alternative to blood transfusion. In order to provide adequate oxygen delivery to tissues, high-viscosity HBOC solutions must have the ability to reversibly bind oxygen (18). Increases in viscosity using these solutions should promote vasodilation *via* mechanical transduction on the endothelium and increase functional capillary density (34), which in turn would lead to an increase in microvascular pressure and subsequently in blood flow. Whether the postulated benefits of artificial blood substitutes with these design constraints are eventually realized and enter routine clinical use hinge on the optimization of ideal viscosity in addition to the restoration of blood volume, but the outlined strategies represent promising steps toward a clinically translatable solution to the ever-growing problem of blood shortage for transfusions in T/HS and other acute and chronic pathologies.

## Author Contributions

CM, FA, KG, PC, and EK reviewed the literature and drafted the manuscript. All authors contributed to the article and approved the submitted version.

## Conflict of Interest

The authors declare that the research was conducted in the absence of any commercial or financial relationships that could be construed as a potential conflict of interest.

## References

[1] Tsai AG Cabrales P Intaglietta M Microvascular perfusion upon exchange transfusion with stored RBCs in normovolemic anemic conditions. Transfusion. (2004) 44:1626–34. 10.1111/j.0041-1132.2004.04128.x 15504169

[2] Saldanha C. Physiological role of erythrocyte nitric oxide. Clin Hemorheol Microcirc. (2016) 64:517–20. 10.3233/CH-168028 27767981

[3] Leiman PG Chipman PR Kostyuchenko VA Mesyanzhinov VV Rossmann MG. Three-dimensional rearrangement of proteins in the tail of bacteriophage T4 on infection of its host. Cell. (2004) 118:419–29. 10.1016/j.cell.2004.07.022 15315755

[4] Liu Y Li D Chen J Xie J Bandyopadhyay S Zhang D. Inhibition of atherogenesis in LDLR knockout mice by systemic delivery of adeno-associated virus type 2-hIL-10. Atherosclerosis. (2006) 188:19–27. 10.1016/j.atherosclerosis.2005.10.029 16300768

[5] Tateishi O Shouda T Sakai T Honda Y Mochizuki S Machida K. Apnea-related heart rate variability in congestive heart failure patients. Clin Exp Hypertens. (2003) 25:183–9. 10.1081/CEH-120019150 12716080

[6] Ghinea N Milgrom E. A new function for the LH/CG receptor: transcytosis of hormone across the endothelial barrier in target organs. Semin Reprod Med. (2001) 19:97–101. 10.1055/s-2001-13916 11394210

[7] Tsai AG Acero C Nance PR Cabrales P Frangos JA Buerk DG. Elevated plasma viscosity in extreme hemodilution increases perivascular nitric oxide concentration and microvascular perfusion. Am J Physiol-Heart Circ Physiol. (2005) 288:H1730–9. 10.1152/ajpheart.00998.2004 15576432

[8] Kimmoun A Novy E Auchet T Ducrocq N Levy B. Hemodynamic consequences of severe lactic acidosis in shock states: from bench to bedside. Crit Care. (2015) 19:175. 10.1186/s13054-015-0896-7 25887061 PMC4391479

[9] Cabrales P Tsai AG Intaglietta M. Microvascular pressure and functional capillary density in extreme hemodilution with low- and high-viscosity dextran and a low-viscosity Hb-based O_2_ carrier. Am J Physiol Heart Circ Physiol. (2004) 287:H363–73. 10.1152/ajpheart.01039.2003 14975932

[10] Pyke KE Tschakovsky ME The relationship between shear stress and flow-mediated dilatation: implications for the assessment of endothelial function. J Physiol. (2005) 568:357–69. 10.1113/jphysiol.2005.089755 16051630 PMC1474741

[11] Doss DN Estafanous FG Ferrario CM Brum JM Murray PA. Mechanism of systemic vasodilation during normovolemic hemodilution. Anesth Analg. (1995) 81:30–4. 10.1213/00000539-199507000-00006 7541185

[12] National Research Council (US) Panel on Statistics for an Aging Population National Research Council Staff Gilford DM Shapiro S Division of Behavioral and Social Sciences and Education Committee on National Statistics The Aging Population in the Twenty-First Century: Statistics for Health Policy. Washington, DC: National Academies Press (US) (1988). Available online at: http://www.ncbi.nlm.nih.gov/books/NBK217737/ (accessed July 20, 2020) 25032446

[13] Gunsilius E Gastl G Petzer AL. Hematopoietic stem cells. Biomed Pharmacother. (2001) 55:186–94. 10.1016/S0753-3322(01)00051-8 11393804

[14] Chen J-Y Scerbo M Kramer G. A review of blood substitutes: examining the history, clinical trial results, and ethics of hemoglobin-based oxygen carriers. Clinics. (2009) 64:803–13. 10.1590/S1807-59322009000800016 19690667 PMC2728196

[15] Buehler PW Mehendale S Wang H Xie J Ma L Trimble CE. Resuscitative effects of polynitroxylated αα-cross-linked hemoglobin following severe hemorrhage in the rat. Free Radic Biol Med. (2000) 29:764–74. 10.1016/S0891-5849(00)00383-X 11053778

[16] Meng F Kassa T Jana S Wood F Zhang X Jia Y Comprehensive biochemical and biophysical characterization of hemoglobin-based oxygen carrier therapeutics: all HBOCs are not created equally. Bioconjug Chem. (2018) 29:1560–75. 10.1021/acs.bioconjchem.8b00093 29570272

[17] S Jahr J Sadighi Akha A Holtby RJ. Crosslinked, polymerized, and PEG-conjugated hemoglobin-based oxygen carriers: clinical safety and efficacy of recent and current products. Curr Drug Discov Technol. (2012) 9:158–65. 10.2174/157016312802650742 21745179

[18] Cabrales P Intaglietta M. Blood substitutes: evolution from non-carrying to oxygen and gas carrying fluids. ASAIO J. (2013) 59:337–54. 10.1097/MAT.0b013e318291fbaa 23820271 PMC3703868

[19] Ray CJ Abbas MR Coney AM Marshall JM. Interactions of adenosine, prostaglandins and nitric oxide in hypoxia-induced vasodilatation: *in vivo* and *in vitro* studies. J Physiol. (2002) 544:195–209. 10.1113/jphysiol.2002.023440 12356892 PMC2290577

[20] Roman RJ. P-450 metabolites of arachidonic acid in the control of cardiovascular function. Physiol Rev. (2002) 82:131–185. 10.1152/physrev.00021.2001 11773611

[21] Chien S. Role of the sympathetic nervous system in hemorrhage. Physiol Rev. (1967) 47:214–88. 10.1152/physrev.1967.47.2.214 5342872

[22] Cannon JW Hemorrhagic shock. N Engl J Med. (2018) 378:370–9. 10.1056/NEJMra1705649 29365303

[23] Chang R Cardenas JC Wade CE Holcomb JB. Advances in the understanding of trauma-induced coagulopathy. Blood. (2016) 128:1043–9. 10.1182/blood-2016-01-636423 27381903 PMC5000842

[24] White NJ Ward KR Pati S Strandenes G Cap AP. Hemorrhagic blood failure: oxygen debt, coagulopathy and endothelial damage. J Trauma Acute Care Surg. (2017) 82:S41. 10.1097/TA.0000000000001436 28328671 PMC5488798

[25] Cohen MJ. Towards hemostatic resuscitation: the changing understanding of acute traumatic biology, massive bleeding, and damage-control resuscitation. Surg Clin North Am. (2018) 92:877–91. 10.1016/j.suc.2012.06.001 22850152

[26] Rhodes RS DePalma RG. Mitochondrial dysfunction of the liver and hypoglycemia in hemorrhagic shock. Surg Gynecol Obstet. (1980) 150:347–52. 7355358

[27] Barbee RW Reynolds PS Ward KR Assessing shock resuscitation strategies by oxygen debt repayment. Shock. (2010) 33:113–22. 10.1097/SHK.0b013e3181b8569d 20081495

[28] Convertino VA Moulton SL Grudic GZ Rickards CA Hinojosa-Laborde C Gerhardt RT. Use of advanced machine-learning techniques for noninvasive monitoring of hemorrhage. J Trauma Acute Care Surg. (2011) 71:S25–32. 10.1097/TA.0b013e3182211601 21795890

[29] Scalea TM Maltz S Yelon J Trooskin SZ Duncan AO Sclafani SJ. Resuscitation of multiple trauma and head injury: role of crystalloid fluids and inotropes. Crit Care Med. (1994) 22:1610–5. 10.1097/00003246-199422100-00017 7924373

[30] Abou-Khalil B Scalea TM Trooskin SZ Henry SM Hitchcock R. Hemodynamic responses to shock in young trauma patients: need for invasive monitoring. Crit Care Med. (1994) 22:633–9. 10.1097/00003246-199404000-00020 8143473

[31] Munoz CJ Lucas A Williams AT Cabrales P. A review on microvascular hemodynamics: the control of blood flow distribution and tissue oxygenation. Crit Care Clin. (2020) 36:293–305. 10.1016/j.ccc.2019.12.011 32172814 PMC7093304

[32] Tsai AG Cabrales P Intaglietta M Mechanisms of oxygen transport in the microcirculation: effects of cell-free oxygen carriers. In: *Blood Substitutes*. San Diego, CA: Elsevier (2006). p. 84–92. 10.1016/B978-012759760-7/50013-5

[33] Cabrales P Intaglietta M Tsai AG. Transfusion restores blood viscosity and reinstates microvascular conditions from hemorrhagic shock independent of oxygen carrying capacity. Resuscitation. (2007) 75:124–34. 10.1016/j.resuscitation.2007.03.010 17481796 PMC3224809

[34] Wettstein R Tsai AG Erni D Winslow RM Intaglietta M. Resuscitation with polyethylene glycol-modified human hemoglobin improves microcirculatory blood flow and tissue oxygenation after hemorrhagic shock in awake hamsters. Crit Care Med. (2003) 31:1824–30. 10.1097/01.CCM.0000069340.16319.F2 12794426

[35] Cabrales P Tsai AG Intaglietta M. Increased plasma viscosity prolongs microhemodynamic conditions during small volume resuscitation from hemorrhagic shock. Resuscitation. (2008) 77:379–86. 10.1016/j.resuscitation.2008.01.008 18308459 PMC3224810

[36] Lipowsky HH Zweifach BW. Application of the two-slit photometric technique to the measurement of microvascular volumetric flow rates. Microvasc Res. (1978) 15:93–101. 10.1016/0026-2862(78)90009-2 634160

[37] Intaglietta M Tompkins WR. Microvascular measurements by video image shearing and splitting. Microvasc Res. (1973) 5:309–12. 10.1016/0026-2862(73)90042-3 4709728

[38] Pittman RN Oxygen transport. In: Nei Granger D, LSU Health Sciences Center, Granger JP, editors. Regulation of Tissue Oxygenation. San Rafael, CA: Morgan & Claypool Life Sciences (2011). p. 19–30. Available online at: https://www.ncbi.nlm.nih.gov/books/NBK54103/ (accessed July 23, 2019). 21634070

[39] Torres Filho IP Intaglietta M. Microvessel PO_2_ measurements by phosphorescence decay method. Am J Physiol-Heart Circ Physiol. (1993) 265:H1434–8. 10.1152/ajpheart.1993.265.4.H1434 8238430

[40] Lucas A Use of Hyperspectral Imaging for the Study of Hemoglobin Oxygen Saturation in the Microcirculation. (2019). Available online at: https://escholarship.org/uc/item/21m251cb (accessed July 10, 2020).

[41] Wettstein R Tsai AG Erni D Lukyanov AN Torchilin VP Intaglietta M. Improving microcirculation is more effective than substitution of red blood cells to correct metabolic disorder in experimental hemorrhagic shock. Shock. (2004) 21:235–40. 10.1097/01.shk.0000114301.36496.ea 14770036

[42] Cabrales P Intaglietta M Tsai AG. Increase plasma viscosity sustains microcirculation after resuscitation from hemorrhagic shock and continuous bleeding. Shock. (2005) 23:549–55. Available online at: https://journals.lww.com/shockjournal/fulltext/2005/06000/increase_plasma_viscosity_sustains.12.aspx 15897809

[43] Villela NR Tsai AG Cabrales P Intaglietta M. Improved resuscitation from hemorrhagic shock with ringer's lactate with increased viscosity in the hamster window chamber model. J Trauma Acute Care Surg. (2011) 71:418–24. 10.1097/TA.0b013e3181fa2630 21248647

[44] Wang P Hauptman JG Chaudry IH. Hemorrhage produces depression in microvascular blood flow which persists despite fluid resuscitation. Circ Shock. (1990) 32:307–18. 2289304

[45] Nakayama S Sibley L Gunther RA Holcroft JW Kramer GC. Small-volume resuscitation with hypertonic saline (2,400 mOsm/liter) during hemorrhagic shock. Circ Shock. (1984) 13:149–59. 6744520

[46] Tsai AG Cabrales P Acharya AS Intaglietta M Resuscitation from hemorrhagic shock: recovery of oxygen carrying capacity or perfusion?: efficacy of new plasma expanders. Transfus Altern Transfus Med. (2007) 9:246–53. 10.1111/j.1778-428X.2007.00086.x

[47] Ye J-M Colquhoun EQ Clark MG. A comparison of vasopressin and noradrenaline on oxygen uptake by perfused rat hindlimb, kidney, intestine and mesenteric arcade suggests that it is in part due to contractile work by blood vessels. Gen Pharmacol Vasc Syst. (1990) 21:805–10. 10.1016/0306-3623(90)91037-R 2276598

[48] Friesenecker B Tsai AG Dunser MW Mayr AJ Martini J Knotzer H. Oxygen distribution in microcirculation after arginine vasopressin-induced arteriolar vasoconstriction. Am J Physiol-Heart Circ Physiol. (2004) 287:H1792–800. 10.1152/ajpheart.00283.2004 15191895

[49] Tsai AG Sakai H Wettstein R Kerger H Intaglietta M An effective blood replacement fluid that targets oxygen delivery, increases plasma viscosity, and has high oxygen affinity. Transfus Altern Transfus Med. (2004) 5:507–13. 10.1111/j.1778-428X.2004.tb00089.x

[50] Fukumura D Jain RK. Role of nitric oxide in angiogenesis and microcirculation in tumors. Cancer Metastasis Rev. (1998) 17:77–89. 10.1023/A:1005908805527 9544424

[51] Williams AT Lucas A Muller CR Bolden-Rush C Palmer AF Cabrales P. Balance between oxygen transport and blood rheology during resuscitation from hemorrhagic shock with polymerized bovine hemoglobin. J Appl Physiol. (2020) 129:97–107. 10.1152/japplphysiol.00016.2020 32552431 PMC7469229

[52] Cooke JP Rossitch E Andon NA Loscalzo J Dzau VJ. Flow activates an endothelial potassium channel to release an endogenous nitrovasodilator. J Clin Invest. (1991) 88:1663–71. 10.1172/JCI115481 1719029 PMC295698

[53] Frangos JA Huang TY Clark CB. Steady shear and step changes in shear stimulate endothelium via independent mechanisms—superposition of transient and sustained nitric oxide production. Biochem Biophys Res Commun. (1996) 224:660–5. 10.1006/bbrc.1996.1081 8713104

[54] Cunningham KS Gotlieb AI. The role of shear stress in the pathogenesis of atherosclerosis. Lab Investig J Tech Methods Pathol. (2005) 85:9–23. 10.1038/labinvest.3700215 15568038

[55] Kim S Ong PK Yalcin O Intaglietta M Johnson PC. The cell-free layer in microvascular blood flow. Biorheology. (2009) 46:181–9. 10.3233/BIR-2009-0530 19581726

[56] Busse R Fleming I. Regulation of endothelium-derived vasoactive autacoid production by hemodynamic forces. Trends Pharmacol Sci. (2003) 24:24–9. 10.1016/S0165-6147(02)00005-6 12498727

[57] Koller A Kaley G. Prostaglandins mediate arteriolar dilation to increased blood flow velocity in skeletal muscle microcirculation. Circ Res. (1990) 67:529–34. 10.1161/01.RES.67.2.529 2115825

[58] Liao JC Hein TW Vaughn MW Huang K-T Kuo L. Intravascular flow decreases erythrocyte consumption of nitric oxide. Proc Natl Acad Sci USA. (1999) 96:8757–61. 10.1073/pnas.96.15.8757 10411948 PMC17589

[59] Lamkin-Kennard KA Jaron DOV Buerk DG. Impact of the fåhraeus effect on NO and O_2_ biotransport: a computer model. Microcirculation. (2004) 11:337–49. 10.1080/10739680490437496 15280073

[60] Maeda N Shiga T Velocity of oxygen transfer and erythrocyte rheology. Physiology. (1994) 9:22–7. 10.1152/physiologyonline.1994.9.1.22

[61] Sriram K Salazar Vazquez BY Yalcin O Johnson PC Intaglietta M Tartakovsky DM. The effect of small changes in hematocrit on nitric oxide transport in arterioles. Antioxid Redox Signal. (2010) 14:175–85. 10.1089/ars.2010.3266 20560785 PMC3014765

[62] Kim S Kong RL Popel AS Intaglietta M Johnson PC. A computer-based method for determination of the cell-free layer width in microcirculation. Microcirculation. (2006) 13:199–207. 10.1080/10739680600556878 16627362

[63] Kim S Kong RL Popel AS Intaglietta M Johnson PC. Temporal and spatial variations of cell-free layer width in arterioles. Am J Physiol Heart Circ Physiol. (2007) 293:H1526–35. 10.1152/ajpheart.01090.2006 17526647

[64] Yalcin O Jani VP Johnson PC Cabrales P. Implications enzymatic degradation of the endothelial glycocalyx on the microvascular hemodynamics and the arteriolar red cell free layer of the rat cremaster muscle. Front Physiol. (2018) 9:168. 10.3389/fphys.2018.00168 29615916 PMC5864934

[65] Yalcin O Choi C Chatpun S Intaglietta M Johnson PC The dependence of cell-free layer thickness in arterioles on systemic hematocrit level. FASEB J. (2009) 23:949 10.1096/fasebj.23.1_supplement.949.7

[66] Cabrales P Nacharaju P Manjula BN Tsai AG Acharya SA Intaglietta M. Early difference in tissue pH and microvascular hemodynamics in hemorrhagic shock resuscitation using polyethylene glycol-albumin- and hydroxyethyl starch-based plasma expanders. Shock. (2005) 24:66–73. 10.1097/01.shk.0000167111.80753.ef 15988323

[67] Tsai AG Vázquez BYS Hofmann A Acharya SA Intaglietta M. Supra-plasma expanders–the future of treating blood loss and anemia without red cell transfusions? J Infus Nurs Off Publ Infus Nurses Soc. (2015) 38:217. 10.1097/NAN.0000000000000103 25871869 PMC5608479

[68] Kerger H Torres Filho IP Rivas M Winslow RM Intaglietta M. Systemic and subcutaneous microvascular oxygen tension in conscious Syrian golden hamsters. Am J Physiol-Heart Circ Physiol. (1995) 268:H802–10. 10.1152/ajpheart.1995.268.2.H802 7864208

[69] Cabrales P Tsai AG Ananda K Acharya SA Intaglietta M. Volume resuscitation from hemorrhagic shock with albumin and hexaPEGylated human serum albumin. Resuscitation. (2008) 79:139–46. 10.1016/j.resuscitation.2008.04.020 18621463 PMC2758307

[70] Cabrales P Tsai AG Intaglietta M. Hemorrhagic shock resuscitation with carbon monoxide saturated blood. Resuscitation. (2007) 72:306–18. 10.1016/j.resuscitation.2006.06.021 17092627

[71] Cabrales P Tsai AG Intaglietta M Hyperosmotic-hyperoncotic vs. hyperosmotic-hyperviscous small volume resuscitation in hemorrhagic shock. Shock. (2004) 22:431–7. 10.1097/01.shk.0000140662.72907.95 15489635

[72] Cabrales P Tsai AG Intaglietta M Is resuscitation from hemorrhagic shock limited by blood oxygen-carrying capacity or blood viscosity? Shock. (2007) 27:380–9. 10.1097/01.shk.0000239782.71516.ba 17414420

[73] Cabrales P Tsai AG Intaglietta M. Polymerized bovine hemoglobin can improve small-volume resuscitation from hemorrhagic shock in hamsters. Shock. (2009) 31:300–7. 10.1097/SHK.0b013e318180ff63 18636045

[74] Cabrales P Tsai AG Intaglietta M. Resuscitation from hemorrhagic shock with hydroxyethyl starch and coagulation changes. Shock. (2007) 28:461–7. 10.1097/shk.0b013e31804880a1 17558350

[75] Kerger H Tsai AG Saltzman DJ Winslow RM Intaglietta M. Fluid resuscitation with O_2_ vs. non-O_2_ carriers after 2 h of hemorrhagic shock in conscious hamsters. Am J Physiol. (1997) 272:H525–37. 10.1152/ajpheart.1997.272.1.H525 9038975

[76] Kerger H Waschke KF Ackern KV Tsai AG Intaglietta M. Systemic and microcirculatory effects of autologous whole blood resuscitation in severe hemorrhagic shock. Am J Physiol. (1999) 276(6 Pt 2):H2035–43. 10.1152/ajpheart.1999.276.6.H2035 10362685

[77] Sakai H Hara H Tsai AG Tsuchida E Johnson PC Intaglietta M. Changes in resistance vessels during hemorrhagic shock and resuscitation in conscious hamster model. Am J Physiol. (1999) 276:H563–71. 10.1152/ajpheart.1999.276.2.H563 9950858

[78] Sakai H Takeoka S Wettstein R Tsai AG Intaglietta M Tsuchida E. Systemic and microvascular responses to hemorrhage shock and resuscitation with Hb vesicles. Am J Physiol Heart Circ Physiol. (2002) 283:H1191–9. 10.1152/ajpheart.00080.2002 12181150

[79] Villela NR Salazar Vazquez BY Intaglietta M. Microcirculatory effects of intravenous fluids in critical illness: plasma expansion beyond crystalloids and colloids. Curr Opin Anaesthesiol. (2009) 22:163–7. 10.1097/ACO.0b013e328328d304 19307891

[80] Wettstein R Cabrales P Erni D Tsai AG Winslow RM Intaglietta M. Resuscitation from hemorrhagic shock with MalPEG-albumin: comparison with MalPEG-hemoglobin. Shock. (2004) 22:351–7. 10.1097/01.shk.0000135253.14076.d9 15377891

[81] Wettstein R Erni D Intaglietta M Tsai AG. Rapid restoration of microcirculatory blood flow with hyperviscous and hyperoncotic solutions lowers the transfusion trigger in resuscitation from hemorrhagic shock. Shock. (2006) 25:641–6. 10.1097/01.shk.0000209532.15317.15 16721273

